# Circular RNA Signature Predicts Gemcitabine Resistance of Pancreatic Ductal Adenocarcinoma

**DOI:** 10.3389/fphar.2018.00584

**Published:** 2018-06-05

**Authors:** Feng Shao, Mei Huang, Futao Meng, Qiang Huang

**Affiliations:** ^1^Medical College, Shandong University, Jinan, China; ^2^Anhui Province Key Laboratory of Hepatopancreatobiliary Surgery, Department of General Surgery, Anhui Provincial Hospital, Hefei, China

**Keywords:** pancreatic ductal adenocarcinoma, gemcitabine resistance, circular RNA, serum marker, diagnosis

## Abstract

Gemcitabine resistance is currently the main problem of chemotherapy for advanced pancreatic cancer patients. The resistance is thought to be caused by altered drug metabolism or reduced apoptosis of cancer cells. However, the underlying mechanism of Gemcitabine resistance in pancreatic cancer remains unclear. In this study, we established Gemcitabine resistant PANC-1 (PANC-1-GR) cell lines and compared the circular RNAs (circRNAs) profiles between PANC-1 cells and PANC-1-GR cells by RNA sequencing. Differentially expressed circRNAs were demonstrated using scatter plot and cluster heatmap analysis. Gene ontology and pathway analysis were performed to systemically map the genes which are functionally associated to those differentially expressed circRNAs identified from our data. The expression of the differentially expressed circRNAs picked up by RNAseq in PANC-1-GR cells was further validated by qRT-PCR and two circRNAs were eventually identified as the most distinct targets. Consistently, by analyzing plasma samples form pancreatic ductal adenocarcinoma (PDAC) patients, the two circRNAs showed more significant expression in the Gemcitabine non-responsive patients than the responsive ones. In addition, we found that silencing of the two circRNAs could restore the sensitivity of PANC-1-GR cells to Gemcitabine treatment, while over-expression of them could increase the resistance of normal PANC-1 and MIA PACA-2 cells, suggesting that they might serve as drug targets for Gemcitabine resistance. Furthermore, the miRNA interaction networks were also explored based on the correlation analysis of the target microRNAs of these two circRNAs. In conclusion, we successfully established new PANC-1-GR cells, systemically characterized the circRNA and miRNA profiles, and identified two circRNAs as novel biomarkers and potential therapeutic targets for Gemcitabine non-responsive PDAC patients.

## Introduction

Pancreatic cancer is one of the most malignant cancers with very poor prognosis. The incidence of pancreatic cancer has been increasing over the past 20 years. 85% of pancreatic cancer is pancreatic ductal adenocarcinoma (PDAC) (Siegel et al., 2017). Although great progress has been made in surgery and other treatments for PDAC, the 5-year survival rate of PDAC is still less than 4%, while the median survival of PDAC patients is only 5–6 months. Since most PDAC patients are diagnosed at an advanced stage, 80% of patients with PDAC is unable to undergo surgical resection treatment. Chemotherapy has become an essential treatment for advanced PDAC (Saung and Zheng, 2017). Gemcitabine is currently a first-line drug of chemotherapy treatment for PDAC patients. However, it is well known that only very few PDAC patients are able to maintain a lasting sensitivity to Gemcitabine-based chemotherapy (Amrutkar and Gladhaug, 2017). The drug resistance has already become the main reason for poor performance of Gemcitabine in the current treatment. Therefore, screening of Gemcitabine resistance related biological markers and improvement of Gemcitabine sensitivity are the main challenge of PDAC research.

Accumulating evidence has shown that circular RNAs (circRNAs), one of endogenous non-coding RNAs, play a key regulatory role in the cellular physiological process and the cancer biological process (Memczak et al., 2013). It is reported that circRNAs act as miRNA sponge which absorb miRNAs and then regulate the expression of miRNA targeted genes (Hansen et al., 2013a,b; Han et al., 2017). Most recently, circRNAs have been shown to be closely associated with various human diseases, such as cancer, cardiovascular disease and neurodegenerative diseases (Westholm et al., 2014; Wang et al., 2016; Chen et al., 2017a; Kristensen et al., 2017). In addition, the inherent stability of circRNAs conferred by the circular structure, allows them to be enriched in the exosomes and stably present in plasma, saliva and other peripheral tissues, which renders them potential diagnostic molecular markers for various diseases (Chen et al., 2017b; Wang et al., 2017).

In this study, we established a new Gemcitabine-resistant cell line (PANC-1-GR) from a pancreatic cancer cell line, PANC-1. The circRNAs expression profile of PANC-1-GR cells was systematically explored comparing with parental PANC-1 cells by RNA sequencing. Our results showed that circRNAs expression profiles are very different between PANC-1 cells and PANC-1-GR cells. The characterization of circRNAs and miRNAs in these cell lines and patient samples led to the identification of two novel circRNAs biomarkers and potential drug targets.

## Materials and Methods

### Patient Samples

This study was approved by the Institutional Ethics Review Board of Anhui Provincial Hospital and was conducted according to the Ethical Guidelines for Human Genome/Gene Research issued by the Chinese Government. The plasma samples of PDAC patients were prospectively collected from 40 patients of Anhui Provincial Hospital from January 2015 to June 2016. All of the patients were histologically or cytologically confirmed as pancreatic ductal adenocarcinoma and received Gemcitabine-based chemotherapy. Twenty of these patients were found to be Gemcitabine non-responsive, as they meet the following definition: progression during or <6 months of previous Gemcitabine treatment including adjuvant therapy.

The peripheral blood (5 ml) was collected in ethylenediaminetetraacetic acid tube, centrifuged at 3000 g for 10 min to harvest plasma, and stored at liquid nitrogen. Patients consent forms were duly signed by the patients according to the regulation of ethical guidelines issued by Anhui provincial hospital.

### Cell Lines

PANC-1 cells were obtained from Shanghai Cell Bank. Cells were cultured in RPMI1640 supplemented in 10% fetal bovine serum (FBS) with 100 U/ml penicillin–100 g/ml streptomycin within a humidified incubator containing 5% CO_2_ at 37°C.

The cell line which was resistant to Gemcitabine was generated by selection under increasing gradient of Gemcitabine in our lab. The initial concentration of Gemcitabine in the cell culture medium was 0.1 μg/ml. The concentration increased when the survival cells entered the logarithmic growth phase. After 40 weeks of continuous Gemcitabine induced culture, the final concentration of Gemcitabine in the resistant cell culture medium reach up to 200 μg/ml. The Gemcitabine resistant cell line was named PANC-1-GR. The PANC-1-GR cell line was cultured in periodically added 10 μg/ml Gemcitabine to maintain cell resistance.

### Cell Viability and Proliferation Assays

MTT assay was performed to determine cell viability of PANC-1 and PANC-1-GR cell lines under Gemcitabine treatment and then indirectly reflected cell sensitivity to Gemcitabine. Cells seeded in 96-well plates, at a density of 5 × 10^4^ cells per well, were given Gemcitabine treatment at different concentrations for 72 h. 100 μl of MTT solution (500 μg/ml) was added to each well and after its conversion to a soluble formazan, cell viability was measured by spectrophotometric absorbance at 570 nm.

Cell proliferation was monitored with xCelligence system. PANC-1 and PANC-1-GR cells were seeded in a 96-well electronic microtiter plate (E-Plate), incubated at 37°C with 5% CO_2_, and then monitored on the RTCA System at 30-min time interval for up to 100 hours. The electronic readout of cell-sensor impedance is displayed in real-time as cell index (CI), which directly influenced by cell attachment, spreading, or cell proliferation. The cell index is presented as mean ± SD from three independent wells (calculated by xCELLigence) (Stefanowicz-Hajduk et al., 2016).

### RNA Extraction and Quality Control

Total RNA was isolated from tumor tissues and plasma samples using TRIzol reagent (Invitrogen, Carlsbad, CA, United States), according to the manufacturer’s protocol. RNA integrity was assessed using standard denaturing agarose gel electrophoresis. The total RNA from each specimen was quantified and quality assurance was provided by NanoDrop ND-1000 spectrophotometer (NanaDrop, Wilmington, DE, United States).

### Transcriptome High-Throughput Sequencing and Subsequent Bioinformatics Analysis

Transcriptome high-throughput sequencing and subsequent bioinformatics analysis were performed by Cloud-Seq Biotech (Shanghai, China). The RNA sequencing data had been deposited in the National Center for Biotechnology Information (NCBI) Gene Expression Omnibus (GEO). The GEO accession number is GSE110580^1^. The scatter plot and cluster heatmap are visualization methods used for assessing the circRNA expression variation. The differentially expressed circRNA between PANC-1 and Gemcitabine resistant PANC-1-GR cells were analyzed by edgeR package in R. Differentially expressed circRNAs with statistical significance (fold changes ≥1.5 and *p* < 0.05) between groups were identified using fold change cut-off or volcano plot filtering, respectively. The Database for Annotation, Visualization and Integrated Discovery (DAVID) bioinformatics tool for KEEG pathway enrichment analysis and Gene Ontology^2^, were applied to determine the roles that these differentially expressed circRNAs played in GO terms of biological pathways (Huang da et al., 2009). The circRNA/microRNA interaction was predicted using Arraystar’s home-made miRNA target prediction software based on TargetScan and miRanda. The circRNA-miRNA network was constructed and visualized using Cytoscape v3.5.1 (Shannon et al., 2003).

### Quantitative Reverse Transcription-Polymerase Chain Reaction Validation Assay

Total RNA samples were reverse-transcribed into cDNA with a random primer using SuperScript^TM^ III Reverse Transcriptase (Invitrogen) according to the manufacturer’s instructions. The expression of circRNAs was measured using quantitative polymerase chain reaction (qPCR) SYBR Green Master Mix (Takara, Tokyo, Japan) in a ViiA 7 Real-time PCR System (Applied Biosystems Inc., Foster City, CA, United States). The sequences of the divergent primers for the detection of the 10 circular RNAs by quantitative reverse transcription-polymerase chain reaction (qRT-PCR) were shown in **Table 2**. The RNA levels were normalized to human GAPDH. The expression levels were analyzed by the 2^-ΔΔCt^ method.

All of the quantitative PCR reactions were conducted in triplicate. The appearance of a single-peak in the melt-curve suggested the specificity of the PCR products.

### Silencing and Over-Expression of circRNA

siRNA sequences for chr14:101402109-101464448+ including: siRNA-1: CUUAAUUGUGGGCUCACAU; siRNA-2: CCUAUAGCUGUGGUAUAAC. siRNA sequences for chr4:52729603-52780244+ including: siRNA-1: CAUAGUAAUAGACGAAUUGA; siRNA-2: UGAAUUCUUAGAAGUUAAAG.

siRNAs were synthesized by GeneChem (Shanghai, China).

The pCD-ciR plasmids was used to carry the circular framework of chr14:101402109-101464448+ and chr4:52729603-52780244+. The primers for chr14:101402109-101464448+ are F: 5^′^-ATAAGTCTACTTTTCTTCCACGTAA-3^′^

R: 5^′^-TTATASTGACATTCTCCTTACTCTGA-3^′^. The primers for chr4:52729603-52780244+ are F: 5^′^-CAGCTGAACTCTCATCTCTCAACAC-3^′^

R:5^′^-CCTTCCAGAAGTTGGCCTCTTAAAC-3^′^.

### Annexin V-FITC Cell Apoptosis Assay

Cells were harvested for Annexin V-Propidium Iodide (PI) staining after 24 h with 0.1 ug/ml gemcitabine treatment. Cells were analyzed by the FACS Calibur (BD Biosciences). Annexin V-FITC^+^ PI-cells were considered as apoptosis cells.

### TUNEL Assay

Cells were exposed to 0.1 ug/ml gemcitabine for 24 h, washed with PBS, and fixed in cold 4% PFA for 30 min, followed by incubation in 0.1% Triton X-100 in PBS for 2 min on ice. After washing twice in PBS, cells were incubated in working solutions from a One-Step TUNEL apoptosis assay kit (Beyotime Biotechnology).

### Statistical Analysis

All experimental data were analyzed using SPSS software (version 22.0; IBM, Armonk, NY, United States) and GraghPad Prism 5.0 (GraphPad Software, La Jolla, CA, United States). The expression level of each circRNA was represented as fold-change using the 2^-ΔΔCt^ method.

## Results

### Comparison of PANC-1 and PANC-1-GR Cell Lines

**FIGURE 1:**
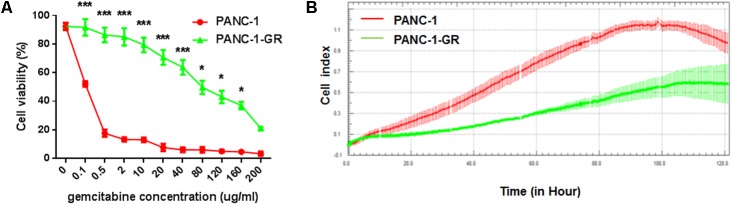
Comparison of PANC-1 and PANC-1-GR cells. **(A)** Gemcitabine cytotoxicity to PANC-1 and PANC-1-GR cells. Cells were incubated continuously with different concentrations of Gemcitabine for 72 h and the cell viability was determined by MTT assay. **(B)** Proliferation assay of PANC-1 and PANC-1-GR cell lines. Data were collected from three independent cultures. Shown are mean values ± standard deviation. ^∗∗∗^*p* < 0.001,^∗^*p* < 0.05.

A new PANC-1-GR cell line was derived from PANC-1 cell line by selecting under Gemcitabine gradients as shown in Section “Materials and Methods.” The drug resistance of this new cell line was confirmed by culturing with Gemcitabine. After incubation with different concentrations of Gemcitabine (0, 0.1, 0.5, 2, 10, 20, 40, 80,120, 160, 200 μg/ml) for 72 h, cell viability was assessed by MTT assay. As shown in cell survival curves in **Figure 1A**, the 50% inhibition concentrations (IC_50_) of Gemcitabine to PANC-1 and PANC-G cells were 0.06 ± 0.003 μg/ml and 56.2 ± 2.16 μg/ml, respectively. Cell proliferating ability of PANC-1 and PANC-1-GR cell lines was further monitored with xCelligence system. Cell growth data showed that PANC-1-GR cells proliferated slower than PANC-1 cells (**Figure 1B**), which may be due to some cell cycle regulatory molecules differentially expressed between parental and resistant lines. And we will investigate the relevant cell cycle regulatory mechanisms in further study. We also determined the expression of the multidrug efflux pump MDR1, which is commonly observed to be upregulated in various drugs resistance cancer cells and also has been shown to cause gemcitabine resistance in pancreatic cancer cells. It was demonstrated that MDR1 expression was up-regulated in PANC-1-GR cells. (Supplementary Figure S1) The results confirmed that a PANC-1-GR cell line was successfully established for subsequent circRNAs profiling.

### Characterization of circRNAs Profiles in PANC-1 and PANC-1-GR Cell Lines

**FIGURE 2:**
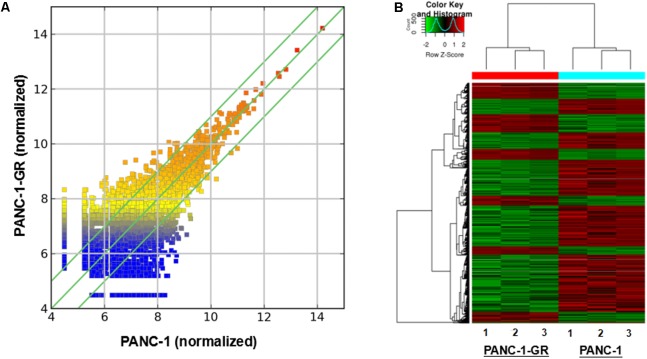
circRNA expression profile of PANC-1-GR cells versus parental PANC-1 cells. **(A)** The scatter plot shows the circRNA expression variation between the parental PANC-1 and PANC-1-GR cell lines. The values of X and Y axes in the scatter plot are the averaged normalized signal values of groups of samples (log2 scaled). The green lines are fold change lines. The circRNAs above the top green line and below the bottom green line indicated more than 1.5-fold change of circRNAs between the two groups of samples. **(B)** Clustered heatmap of the differentially expressed circRNAs in three paired PANC-1 and PANC-1-GR cell lines. Rows represent circRNAs while columns represent cell lines. The circRNAs were classified according to the Pearson correlation.

To screen circRNAs which could be involved in Gemcitabine resistance in PDAC, we analyzed and compared circRNAs expression in PANC-1 cells and PANC-1-GR cells using transcriptome high-throughput sequencing analysis. Total RNAs were isolated from PANC-1 and PANC-1-GR cell lines and analyzed by RNA sequencing. Differential gene expression analysis between PANC-1 and PANC-1-GR cells revealed 126 circRNAs whose expression was significantly different in these two cell lines (fold change ≥2.0, *p* ≤ 0.05), with 68 of them up-regulated and 58 down-regulated in PANC-1-GR cells compared to PANC-1 cells (**Figure 2**).

### CircRNAs Gene Symbols and Pathway Analysis

**FIGURE 3:**
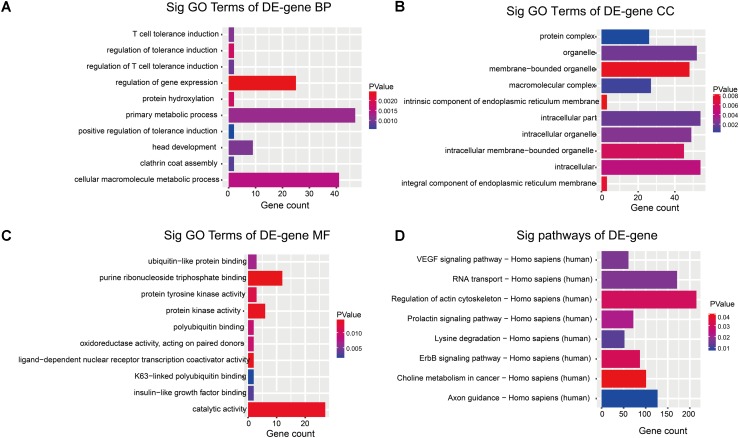
GO enrichment and pathway analysis for dysregulated circRNAs gene symbols. **(A)** Most significantly enriched GO [–log10 (*P* value)] terms of circRNAs gene symbols according to biological process. **(B)** Most significantly enriched [–log10 (*P* value)] GO terms of circRNAs gene symbols according to cellular component. **(C)** Most significantly enriched [–log10 (*P* value)] GO terms of circRNAs gene symbols according to molecular function. **(D)** The bar plot shows the top 10 enrichment score [–log10 (*P* value)] of the significantly enriched pathways.

Recent studies have shown that circRNAs are derived from the exons or introns of their parental genes and may regulate the expression of the parental genes (Lasda and Parker, 2014). Based on evaluation of the parental genes attribute in the biological process, cellular components and molecular functions and pathways, we conducted GO and pathway analysis for circRNAs to speculate their potential functions. The lower the *p* value was, the more significant the correlation was (*p* < 0.05 is recommended). We found that the most significantly enriched GO term in the biological process was the positive regulation of tolerance induction (GO:0002645, *P* = 0.0005) (**Figure 3A**); the most significantly enriched GO term in the cellular component was protein complex (GO:0043234, *P* = 0.0001) (**Figure 3B**); the most significantly enriched GO term in the molecular function was K63-linked polyubiquitin binding (GO:0070530, *P* = 0.0016) (**Figure 3C**). Among the significantly related eight pathways, ErbB signaling pathway and VEGF signaling pathway were previously reported to be involved in the progression of PDAC (**Figure 3D**).

### Quantitative PCR Validation in Cell Lines

**FIGURE 4:**
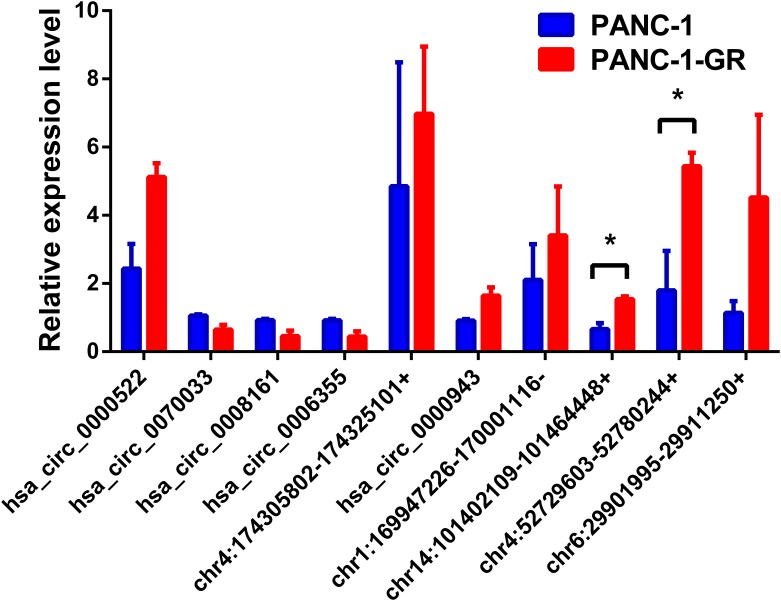
Validation of the top 10 dysregulated circRNAs by quantitative RT-PCR analysis. The top 10 most differentially expressed circRNAs were validated by qRT-PCR in PANC-1 and PANC-1-GR cell lines. The results are presented as mean ± SEM. ^∗^*p* < 0.05.

To verify the sequencing results, the top 10 most differentially expressed circRNAs in PANC-1 and PANC-1-GR cells including seven up-regulated circRNAs (hsa_circ_0000522, hsa_circ_0070033, hsa_circ_0000943, chr1:169947226-170001116-, chr14:101402109-101464448+, chr4:52729603-52780244+, and chr6:29901995-29911250+) and three down-regulated circRNAs (hsa_circ_0070033, hsa_circ_0008161, and hsa_circ_0006355) (**Table 1**) were further confirmed by qRT-RCR. The qRT-PCR data showed that although the trend of expression patterns of these 10 circRNAs were consistent with the sequencing results, among these circRNAs, only two of them (chr14:101402109-101464448+, chr4:52729603-52780244+) were found to be the most significantly up-regulated in PANC-1-GR cell line (**Figure 4**).

**Table 1:** Top 10 most differentially expressed circRNAs between PANC-1 and PANC-1-GR.

circRNA	chrom	txStart	txEnd	Strand	CircBase ID	Gene Name	Regulation
1	chr14	21825356	21829372	-	hsa_circ_0000522	SUPT16H	Up
2	chr4	77045802	77065626	-	hsa_circ_0070033	NUP54	Down
3	chr1	15537793	155385714	-	hsa_circ_0008161	ASH1L	Down
4	chrX	154736558	154766779	-	hsa_circ_0006355	TMLHE	Down
5	chr4	174305801	174325101	+	Novel	SCRG1	Up
6	chr19	47421744	47440665	+	hsa_circ_0000943	ARHGAP35	Up
7	chr1	169947225	170001116	-	Novel	KIFAP3	Up
8	chr14	101402108	101464448	+	Novel	SNORD114-1	Up
9	chr4	52729602	52780244	+	Novel	DCUN1D4	Up
10	chr6	29901994	29911250	+	Novel	HLA-G	Up

**Table 2:** Primers used for qRT-PCR analysis of circular RNA and mRNA levels.

CircRNA Name	Primer sequences, 5^′^-3^′^	Tm (°C)	PS (bp)
hsa_circ_0000522	F: ACTTTGAGCGGGTCCAGTTT	61.05	195
	R: TCTGAAGGTTCAAGGCTGGT	59.84	
hsa_circ_0070033	F: GCCAAAATTGCACAATACAAGA	60.01	198
	R: TTGTGCCAAAACCAGTACCA	60	
hsa_circ_0008161	F: TTGGCTTAGTTGGATCCTCTG	59.32	198
	R: TTTTCCCTTGGGATGAGAGA	59.6	
hsa_circ_0006355	F: AGGCACCTGAGGAATTTGAA	59.67	195
	R: TCCTTTCTCCTGCCACATTC	60.2	
chr4:174305802-174325101+	F: GCTGTTTCACAGACACAAGCA	60.09	161
	R: CCCACGTTACTGAGCACAAA	59.76	
hsa_circ_0000943	F: GACAGAAACCAAAGCCCAAA	60.09	197
	R: TGGTCACTGTTCACCACCTC	59.55	
chr1:169947226-170001116-	F: ACCAGATGGTTTTCCACCAA	60.21	176
	R: CTTTGTTGCTTTCCTCATTGC	59.87	
chr14:101402109-101464448+	F: CAGGATGGGTAGACCAGAGC	59.68	182
	R: TACCCCACGGATCTAACTGC	59.96	
chr4:52729603-52780244+	F: TGGCATTTCTAGTCCCTTTTT	57.88	184
	R: TGCCAGTGTTGAGAGATGAGA	59.56	
chr6:29901995-29911250+	F: AAGGATTACATCGCCCTGAA	59.53	198
	R: GTCCCTGGTACAGGTGTGCT	60.03	
GAPDH	F: GGCCTCCAAGGAGTAAGACC	60.07	122
	R: AGGGGAGATTCAGTGTGGTG	59.96	

*Tm, melting temperature; PS, product size; bp, base pairs; F, Forward; R, Reverse*

### Expression of circRNAs in Plasma of PDAC Patients

**FIGURE 5:**
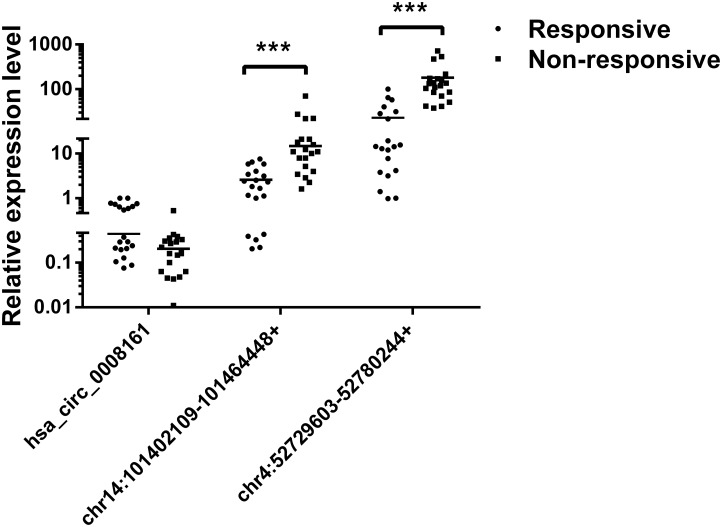
The expression levels of circRNAs in plasma of PDAC patients. PDAC patients who had received chemotherapy were divided into responsive and non-responsive groups according to the effect of Gemcitabine treatment. Plasma from these patients were collected and analyzed by qRT-PCR for three circRNAs, *n* = 20. Data are presented as fold changes of expression levels to GAPDH. ^∗∗∗^*p* < 0.001.

Subsequently, we verified the expression levels of these two most significant circRNAs (chr14:101402109-101464448+, chr4:52729603-52780244+) in plasma of PDAC patients who received Gemcitabine-based chemotherapy via qRT-PCR. In addition, hsa_circ_0008161, which was not significantly regulated as in qRT-PCR data, was used as a negative control. The Gemcitabine-treated PDAC patients were divided into responsive and non-responsive groups according to the effect of Gemcitabine treatment. The clinical characteristics of the patients were shown in **Table 3**. Consistent to RNA sequencing and qRT-PCR data from PANC-1-GR cells, chr14:101402109-101464448+ and chr4:52729603-52780244+ were found significantly up-regulated in non-responsive group (*p* < 0.001), while there was no significant difference in hsa_circ_0008161 (**Figure 5**).

**Table 3:** Clinical characteristics of the patients.

Clinicopathologic factors	*n*	Responsive	Non-responsive	*P*-value
Age				0.519
<60 years	24	11	13	
≥60 years	16	9	7	
Sex				0.256
Male	31	14	17	
Female	9	6	3	
Tumor location				0.376
Head	34	18	16	
Body/tail	6	2	4	
Serum CA19-9				0.633
≤37 U/mL	5	3	2	
>37 U/mL	35	17	18	
Number of metastatic lesions				0.376
1	6	2	4	
≥2	34	18	16	
Karnofsky performance status score				0.248
100	7	4	3	
90	8	3	5	
80	19	10	9	
70	3	2	1	
50–60	3	1	2	

### Network of circRNAs and the Predicted Binding miRNAs

**FIGURE 6:**
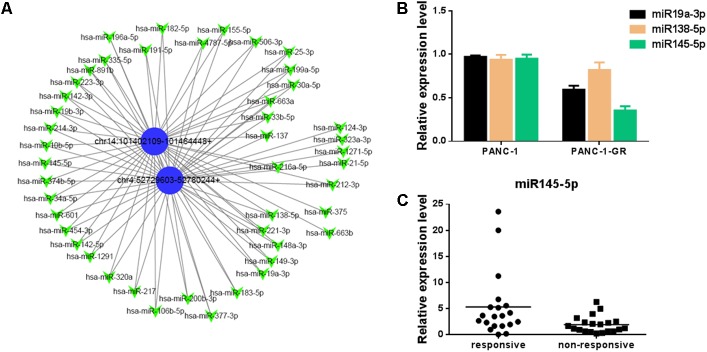
Network of circular RNAs and the predicted binding miRNAs. **(A)**The two circRNAs biomarkers were annotated in detail according to the circRNA/miRNA interaction information by Cytoscape. **(B)** miR19a-3p, miR138-5p and miR145-5p expression in PANC-1 and PANC-1-GR cells. **(C)** miR145-5p expression in plasma of PDAC patients who received gemcitabine treatment, including responsive and non-responsive groups.

To better explore and understand the upstream and downstream miRNAs associated to the two circRNAs, we analyzed the potential binding miRNAs for the two circRNAs by sequence analysis with TargetScan. A tree diagram of circRNAs and their potential binding miRNAs is generated in **Figure 6A**. Based on circRNA/miRNA interaction network, chr14:101402109-101464448+ and chr4:52729603-52780244+ were predicted to be able to bind a spectrum of miRNAs with known functions, suggesting their potential roles in Gemcitabine resistance of PDAC. We selected three potential target miRNAs from the tree diagram, including miR-19a-3p, miR-138-5p, and miR-145-5p, which may bind both of the two circRNAs, and compare the expression of them between PANC-1 and PANC-1-GR cells. It was found that miR-19a-3p and miR-145-5p were down-regulated in PANC-1-GR cells, while miR-138-5p expression did not change significantly (**Figure 6B**). We also analyzed the miR-145-5p expression of plasma of PDAC patients who received gemcitabine treatment. It was found miR-145-5p was down-regulated in non-responsive group, compared with responsive group (**Figure 6C**).

### Silencing of circRNAs Enhances Gemcitabine Sensitivity of PANC-1-GR

**FIGURE 7:**
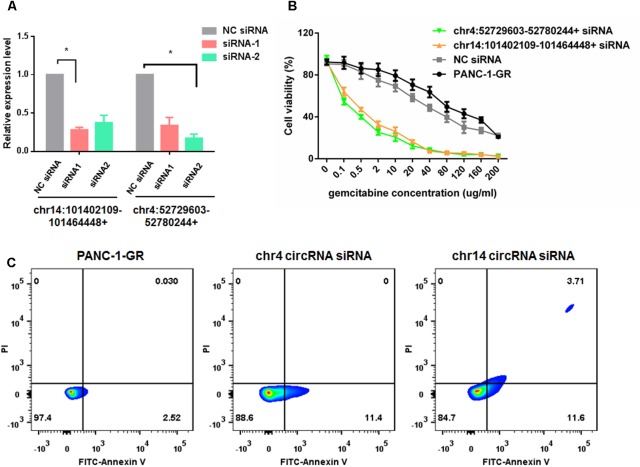
Silencing of circRNAs enhances Gemcitabine sensitivity of PANC-1-GR cell lines. **(A)** qRT-PCR analysis of circRNA expression level after siRNAs transfection. NC siRNA: negative control siRNA. **(B)** Cell viability of PANC-1-GR cells after transfection with siRNAs. Cells were incubated with different concentrations of Gemcitabine for 72 h and then cell viability was determined by MTT assay. ^∗^*p* < 0.05. **(C)** Annexin V-FITC staining assay was performed for PANC-1-GR cells and siRNA tranfected PANC-1-GR cells.

To evaluate the functions of these two new circRNAs in Gemcitabine resistance, we applied small RNA interference (siRNAs) to silence the expression of chr14:101402109-101464448+ and chr4:52729603-52780244+ in PANC-1-GR cells. Two siRNAs were designed to target the backsplice sequence of each circRNA, respectively. A non-specific control siRNA sequence was also used as the negative control. After transfection, siRNA1 of chr14:101402109-101464448+ and siRNA2 of chr4:52729603-52780244+, dramatically inhibited the expression of chr14:101402109-101464448+ and chr4:52729603-52780244+, respectively, in PANC-1-GR cells (**Figure 7A**), which were further used for transfection in cytotoxicity assay. It was demonstrated that after silencing of chr14:101402109-101464448+ or chr4:52729603-52780244+, PANC-1-GR cells restored sensitivity to Gemcitabine (**Figure 7B**). Annexin V staining apoptosis assay also demonstrated that siRNA group had more Annexin V positive apoptosis cells after cells were cultured with 0.1 ug/ml gemcitabine for 24 h (**Figure 7C**). These results suggested that the two circRNAs may serve as potential therapeutic targets for Gemcitabine resistance in PDAC.

### Overexpression of circRNAs Enhances Gemcitabine Resistance of PANC-1 and MIA PACA-2 Cells

**FIGURE 8:**
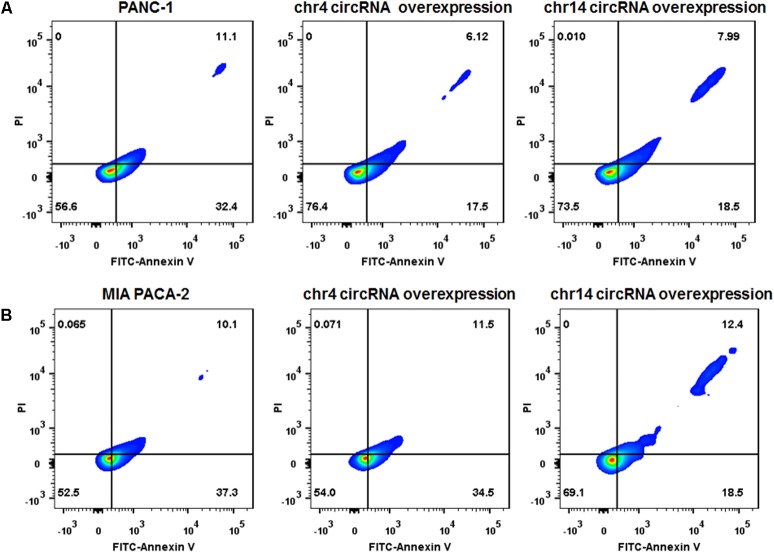
Over-expression of circRNAs enhances Gemcitabine resistance of PANC-1 and MIA PACA-2 cells. **(A)** Annexin V-FITC staining assay was performed for PANC-1 cells, chr4 circRNA and chr14 circRNA tranfected PANC-1 cells. Cells were incubated with 0.1 ug/ml of Gemcitabine for 24 h. **(B)** Annexin V-FITC staining assay was performed for MIA PACA-2 cells, chr4 circRNA tranfected and chr14 circRNA tranfected MIA PACA-2 cells. Cells were incubated with 0.1 ug/ml of Gemcitabine for 24 h.

We also overexpressed the circRNAs in parental PANC-1 and MIA PACA-2 cells. pCD-ciR plasmids was used to carry the circular framework of chr14:101402109-101464448+ and chr4:52729603-52780244+. After transfection and over-expression of chr14:101402109-101464448+ and chr4:52729603-52780244+ in PANC-1 cells, PANC-1 cells showed more resistant to gemcitabine, with less Annexin V positive cells after 0.1 ug/ml gemcitabine treatment for 24 h (**Figure 8A** and Supplementary Figure S2). The same result can be observed in MIA PACA-2 cells, when tranfected with chr14:101402109-101464448+ circular framework (**Figure 8B** and Supplementary Figure S2). However, only overexpression of chr14:101402109-101464448+ was able to exert some resistant phenotype to Gemcitabine in MIA PACa-2 while chr4:52729603-52780244+ was not. The downstream regulatory function of chr4:52729603-52780244+ may be compensated or antagonized by other regulatory mechanisms in MIA PACA-2 cells. We also analyzed miR-145 expression in parental PANC-1 and MIA PACA-2 cells when they overexpressed chr4:52729603-52780244+ and chr14:101402109-101464448+. It was found that miR-145 expression decreased to different extents, when chr4:52729603-52780244+ and chr14:101402109-101464448+ overexpressed in parental PANC-1 and MIA PACA-2 cells. (Supplementary Figure S3) It further suggests that miR145 may be involved in gemcitabine resistance by circRNA-miRNA interaction.

## Discussion

In this study, we developed Gemcitabine resistant pancreatic cancer cell line PANC-1-GR as a research tool to investigate Gemcitabine resistance. Subsequently, we compared the differences of circRNAs expression profile between PANC-1 and PANC-1-GR cell lines using RNA sequencing analysis. From the sequencing data, it was found that there were 68 up-regulated circRNAs and 58 down-regulated circRNAs which are possibly related to Gemcitabine resistance in PANC-1-GR. Upon validating the top 10 dys-regulated circRNAs using qRT-PCR in these cell lines, it is interesting to see that RNA sequencing data was quite consistent with qRT-PCR and combining both, we were able to identify two most distinctly expressed circRNAs from PANC-1-GR cells when compared to PANC-1 cells. It is even more exciting to confirm that these two markers are also consistently found to be highly expressed in the plasma from Gemcitabine non-responsive PDAC patients but not in Gemcitabine responsive ones. Our study demonstrated that the two circRNAs may be functionally involved in generating Gemcitabine resistance as the silencing of them can restore the sensitivity of PANC-1-GR cells to Gemcitabine.

Besides, GO and pathway analysis of circRNAs patent genes were investigated. GO enrichment analysis revealed that some gene symbols were involved in the regulation of biological process, cellular component and molecular function. Among the GO terms found in this study, “primary metabolic process” and “insulin-like growth factor binding” may play important roles in the drug resistance of PANC-1 cells via drug metabolic or cell proliferation pathway (Ireland et al., 2016; Chen et al., 2017c). Meanwhile, “ErbB signaling pathway” has been reported to be involved in Gemcitabine resistance of pancreatic cancer (Skrypek et al., 2015) and “VEGF signaling pathway” has been reported to be involved in the progression of pancreatic cancer, which may contribute to cell drug resistance (Zhou et al., 2016; Zhu et al., 2016). These findings indicated that inaddition to the two biomarkers identified in this study, there could be more circRNAs involved in the Gemcitabine resistance of PANC-1-GR cells. Recent studies have demonstrated that circRNAs could regulate gene expression as miRNA sponges or potent competitive endogenous RNA (ceRNA) molecules (Qu et al., 2017). Given that miRNAs play important roles in the Gemcitabine resistance of pancreatic cancer (Amponsah et al., 2017; Chaudhary et al., 2017; Xiong et al., 2017), some circRNAs could likely be involved in Gemcitabine resistance via interacting with miRNAs. We found that the majority of circRNAs contained one or more miRNA binding sites based on the sequences analysis. Since we identify the two circRNAs markers in this study, preparing more information about miRNA networks of these two circRNAs is meaningful as it may lead to a better understanding of their upstream and downstream miRNA targets which could serve as potential markers and drug targets. The association of miRNAs with PDAC indicated that circRNAs may have a regulatory role in PDAC. For example, among the founded potential circRNA/miRNA interactions, chr4:52729603-52780244+ is potentially able to bind miR124-3p, which has been reported playing a critical role in Gemcitabine resistance of pancreatic cancer (Li et al., 2016). MiR-145, which may bind to both chr14:101402109-101464448+ and chr4:52729603-52780244+, was also known to be associated with the resistance of pancreatic cancer cells to Gemcitabine (Zhuang et al., 2017). It was found that MiR-145-5p was down-regulated in both PANC-1-GR cells and plasma of non-responsive patients. Certainly, future studies are required to clarify the underlying mechanism of these circRNAs-miRNA interactions in Gemcitabine resistance of pancreatic cancer.

In conclusion, our study provides a new research tool, PANC-1-GR cell line and based on this tool, new insights into Gemcitabine resistance in pancreatic cancer treatment by identifying two novel circRNAs biomarkers and drug targets. Future investigation should be followed up focusing on the exploration of underlying mechanism of the two circRNAs and their associated networks. We hope this work would help to accelerate the development of novel therapeutic strategies targeting Gemcitabine-based chemotherapy of PDAC patients.

## Author Contributions

FS and MH carried out the experiments and drafted the initial manuscript. FM contributed to the literature search and bioinformatic analysis. QH reviewed the statistics and contributed to critical revisions. All authors reviewed and approved the final manuscript as submitted.

## Conflict of Interest Statement

The authors declare that the research was conducted in the absence of any commercial or financial relationships that could be construed as a potential conflict of interest.

## Footnotes

1 www.ncbi.nlm.nih.gov/geo/query/acc.cgi?acc=GSE110580

2 https://david.ncifcrf.gov/
