# Supplementary material for: Circular RNA Signature Predicts Gemcitabine Resistance of Pancreatic Ductal Adenocarcinoma
Source: Front Pharmacol. 2018 Jun 5;9:584. doi: 10.3389/fphar.2018.00584 (PMC5996282; doi:10.3389/fphar.2018.00584)
Supplement: Supplementary file 1 [file Presentation_1.PDF]

Supplementary Material

Circular RNA signature predicts Gemcitabine resistance of pancreatic ductal adenocarcinoma

Feng Shao*, Mei Huang*, Futao Meng, Qiang Huang

*** Correspondence:** Qiang Huang: ahslyyhq2016@163.com

1.1 Supplementary Figures

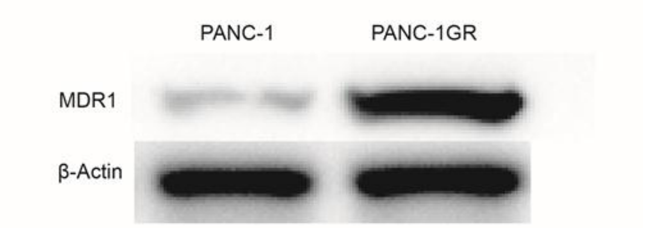

Supplementary Figure 1. Western blot analysis of MDR1 expression in PANC-1 and PANC-1-GR cells.

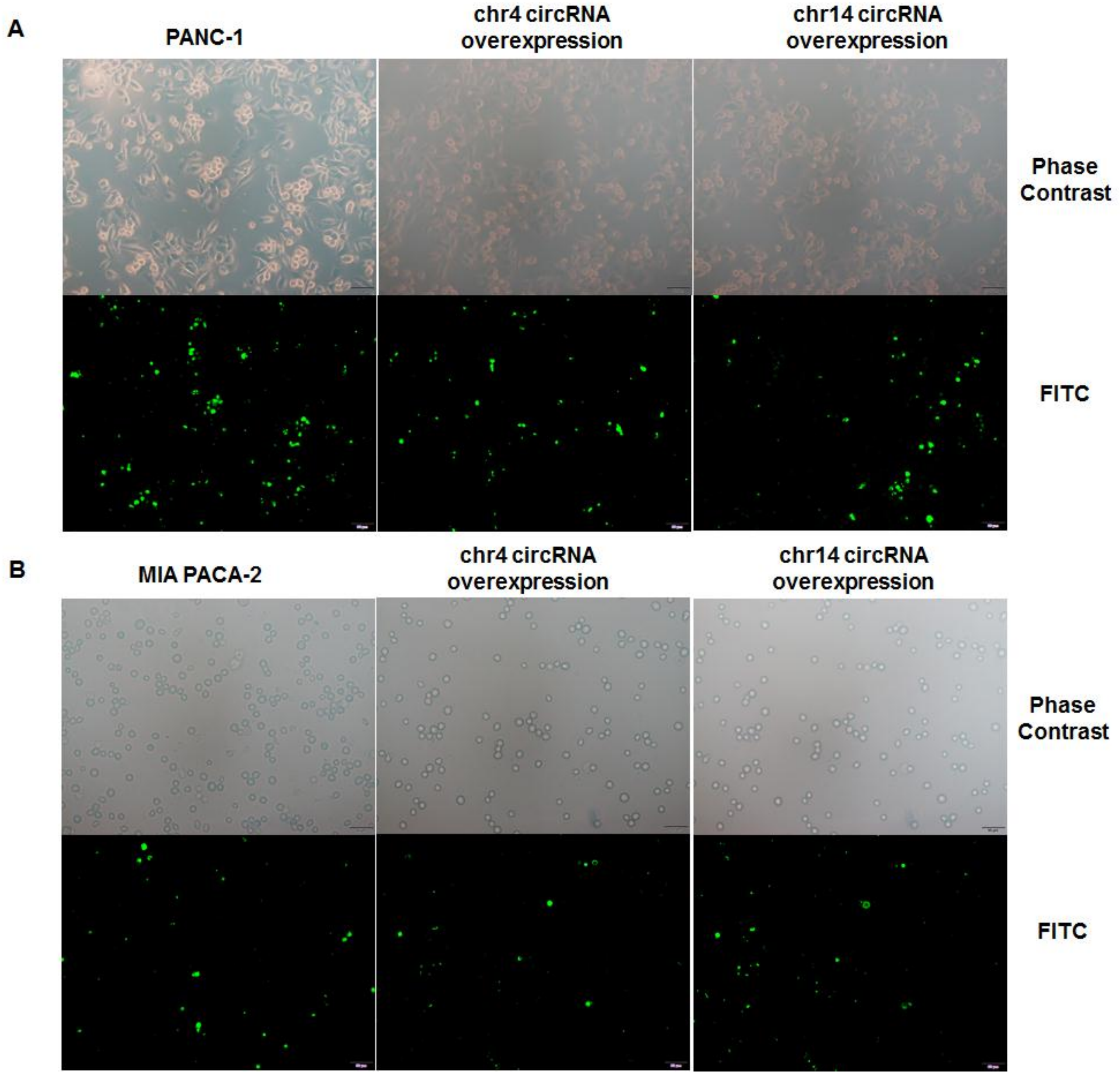

Supplementary Figure 2. TUNEL analysis of PANC-1 and MIA PACA-2 cells. (A) PANC-1 cells were tranfected with circular framework of chr14:101402109-101464448+ and chr4:52729603-52780244+. (B) MIA PACA-2 cells were tranfected with circular framework of chr14:101402109-101464448+ and chr4:52729603-52780244+.

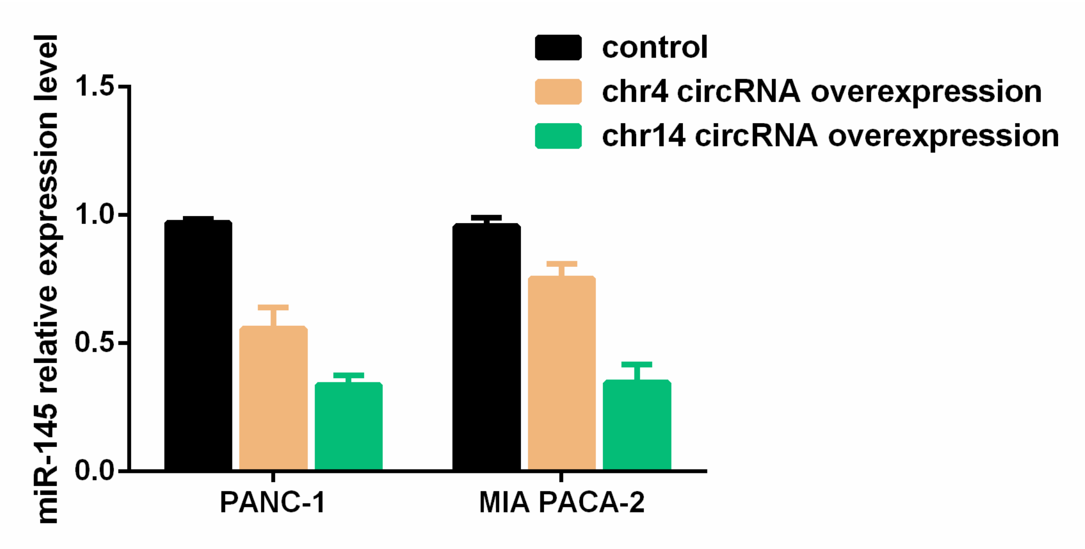

Supplementary Figure 3. Quantitative PCR analysis of miR-145 expression in PANC-1 and MIA PACA-2 cells after overexpression of circRNAs.
